# Potential association between dementia polygenic risk scores and subjective cognitive burden in a post-COVID cohort: a hypothesis-generating study

**DOI:** 10.3389/fpubh.2026.1867492

**Published:** 2026-10-01

**Authors:** Aleemah Nasif, Punya Sunil, Ranajit Das

**Affiliations:** 1 Centre for Systems Biology and Molecular Medicine, Yenepoya Research Center, Yenepoya (Deemed to be University), Mangalore, Karnataka, India; 2 Centre for Integrative Research in Clinical Genetics and Metabolic Medicine, Yenepoya Medical College, Yenepoya (Deemed to be University), Mangalore, Karnataka, India

**Keywords:** COVID-19, dementia risk, GWAS, human genomics, polygenic risk score, post-COVID cognitive symptoms, South Asian population, subjective cognitive decline

## Abstract

**Background:**

Coronavirus disease 2019 (COVID-19) may be associated with persistent subjective cognitive complaints, including difficulties with attention, memory, and executive function, in some individuals with post-acute sequelae of SARS-CoV-2 infection (PASC, “long COVID”). Inter-individual variability raises the possibility that host genetic factors contribute to susceptibility. This study explored the relationship between dementia-related polygenic risk scores (PRS) and subjective cognitive burden after COVID-19.

**Methods:**

Genome-wide genotype data from 60 individuals with a history of COVID-19 were merged with 915 South Asian population reference samples (total *N* = 975). Dementia-related PRS were generated using PRSice-2 across prespecified GWAS *p*-value thresholds. A subset of 15 participants completed a structured, study-specific self-report questionnaire assessing perceived cognitive difficulties across multiple domains 24–30 months after infection. Principal component analysis (PCA) summarized shared variance across cognitive domains. Associations between PRS and cognitive scores were examined using Spearman’s rank correlation. All analyses were considered exploratory because no independent validation cohort was available.

**Results:**

COVID-19–affected individuals aged 18–52 years showed a modest rightward shift in dementia-related PRS compared with population reference samples, with substantial overlap between groups (mean standardized PRS 0.24 vs. − 0.01; Mann–Whitney U = 29,504.5, *p* = 0.065; Cohen’s d = 0.25). Memory-related difficulties contributed most strongly to the shared variance across cognitive domains, with PC1 explaining 52.5% of the variance. Among the 15 participants with cognitive assessment, PRS showed a positive but uncertain association with subjective cognitive burden (Spearman’s *r* = 0.51, 95% CI − 0.02 to 0.82, *p* = 0.054).

**Conclusion:**

This hypothesis-generating study identified a possible relationship between dementia-related genetic liability and subjective cognitive burden after COVID-19. The PRS group comparison was descriptive and should not be interpreted as evidence of increased dementia risk. The phenotype–genotype finding is severely limited by the small subgroup, possible selection bias, non-validated subjective assessment, lack of multivariable adjustment, and cross-sectional design. Validation in larger, ancestry-matched, longitudinal cohorts is required.

## 1 Introduction

Coronavirus disease 2019 (COVID-19), caused by severe acute respiratory syndrome coronavirus 2 (SARS-CoV-2), is increasingly recognized as a multisystem disorder with persistent neurological and cognitive sequelae extending beyond the acute phase of infection. A substantial proportion of individuals recovering from COVID-19 report ongoing cognitive complaints, including difficulties with memory, attention, learning, and executive functioning, even following mild or non-hospitalized disease (1, 2). These post-COVID cognitive symptoms, commonly described within the spectrum of post-acute sequelae of SARS-CoV-2 infection (PASC), show marked inter-individual variability, suggesting that host-specific biological factors may influence susceptibility to cognitive manifestations.

Human cognitive traits such as memory and learning exhibit substantial inter-individual variability and are influenced by both environmental and genetic factors. Large-scale genome-wide association studies (GWAS) have demonstrated that general cognitive ability and domain-specific cognitive functions are heritable and shaped by numerous common genetic variants of small effect distributed across the genome, supporting a highly polygenic architecture (3–5). GWAS meta-analyses have further identified multiple loci associated with verbal short-term memory and learning performance, underscoring the genetic complexity underlying memory-related traits (6). Family- and SNP-based heritability studies confirm that memory and related cognitive traits retain a substantial genetic component even after accounting for major risk loci, indicating that additional variants contribute to cognitive variability (7).

Beyond single-variant associations, integrative genetic analyses have shown that general cognitive function, encompassing memory-related processes, displays SNP-based heritability enriched within evolutionarily conserved genomic regions, suggesting shared biological pathways underlying cognitive abilities (8). Polygenic risk scores (PRS), which aggregate the effects of many genetic variants weighted by GWAS-derived effect sizes, provide a quantitative framework for estimating cumulative genetic susceptibility. PRS have been applied to study individual differences in cognitive performance and educational attainment, as well as genetic vulnerability to neurodegenerative conditions such as Alzheimer’s disease and related dementias (9–11). The PRS used in the present study was derived from the FinnGen all-cause dementia phenotype (finn-b-KRA_PSY_DEMENTIA), rather than an Alzheimer’s disease-specific or neuropathologically defined dementia phenotype. This source phenotype was generated predominantly in European-ancestry participants and includes heterogeneous clinical dementia diagnoses (12). Accordingly, the resulting score should be interpreted as a research measure of inherited susceptibility associated with an all-cause dementia phenotype, not as a measure of clinical dementia risk or near-term dementia risk in this relatively young South Asian cohort. The use of a European-derived base GWAS also limits transferability of effect sizes to this cohort and is an important consideration when interpreting the PRS (13).

Systemic infections and inflammatory insults have been proposed as potential modifiers of cognitive trajectories, and emerging evidence suggests that COVID-19 may act as a biological stressor capable of exacerbating or unmasking latent cognitive vulnerabilities (2, 14, 17). In this context, genetic susceptibility to cognitive decline may interact with post-infectious processes to influence subjective cognitive experiences following COVID-19. However, the relationship between dementia-related polygenic risk and self-reported cognitive difficulties in post-COVID populations remains poorly explored, particularly in South Asian populations (Figure 1).

**Figure 1:**
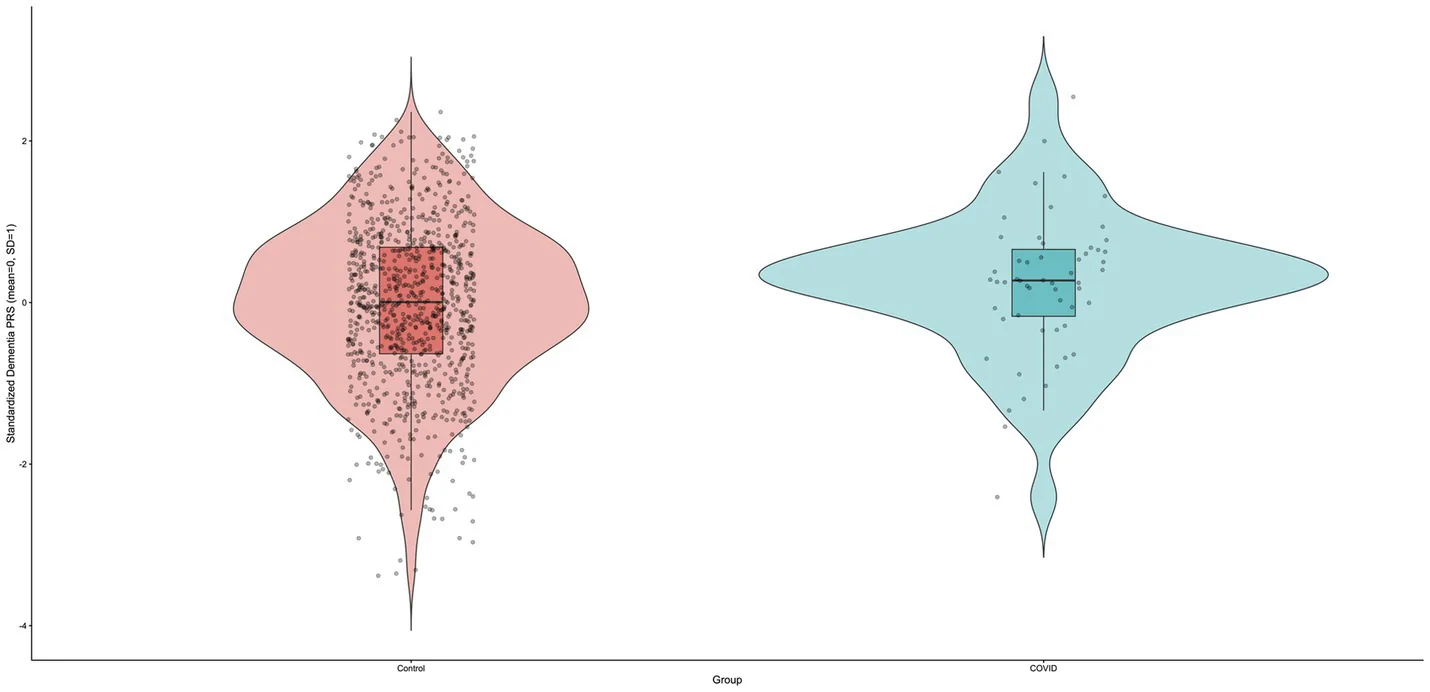
Comparison of standardized polygenic risk scores (PRS) for dementia between COVID-19–affected individuals and South Asian population reference samples. The distributions show substantial overlap with a modest rightward shift among participants with a history of COVID-19 infection. This figure is descriptive and should not be interpreted as evidence of increased dementia risk or as a case–control genetic association, because the reference samples were not matched clinical controls and residual population-stratification differences may remain.

Given the highly polygenic nature of cognitive traits and the growing burden of post-COVID cognitive complaints, exploratory integration of GWAS-derived PRS with cognitive phenotypes may provide insights into genetic contributions to post-infectious cognitive variability. In this brief research report, we present a hypothesis-generating analysis examining whether polygenic risk for dementia is associated with subjective cognitive burden in individuals with a history of COVID-19 infection (Figure 2).

**Figure 2:**
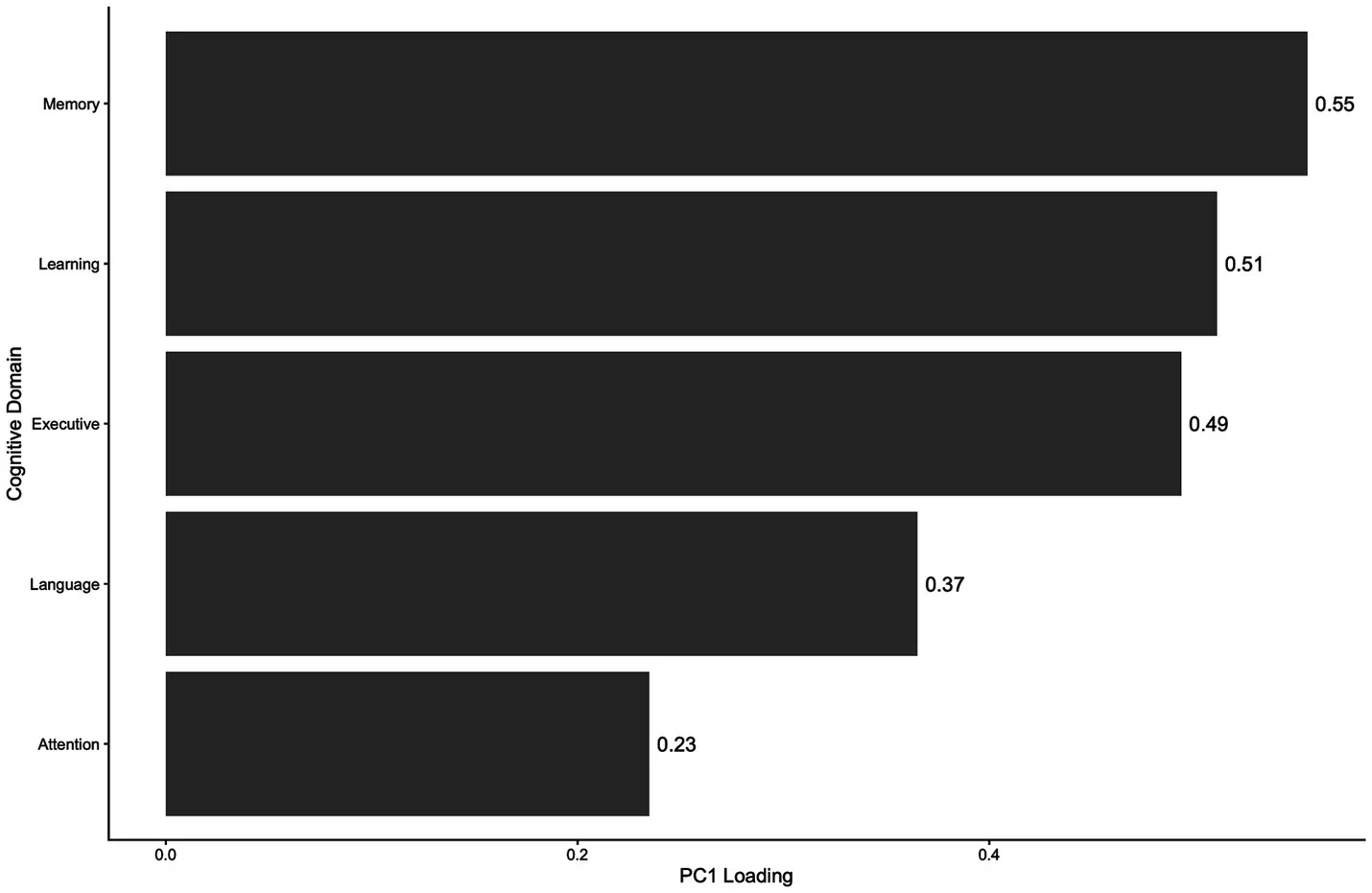
Exploratory PCA loadings for five self-reported cognitive domains. PC1 was derived from memory, executive function, attention, learning, and language domain scores. Memory showed the largest loading in this sample. Because the PCA was performed in *n* = 15 participants using a study-specific, non-validated questionnaire, the loading pattern is descriptive and should not be interpreted as evidence of a validated latent clinical construct.

## 2 Materials and methods

All analyses were conducted as exploratory, hypothesis-generating investigations. Dementia-related PRS were used as a proxy for cumulative genetic vulnerability to cognitive decline rather than as indicators of clinical dementia. PCA was applied as a dimensionality-reduction approach to summarize shared variance across self-reported cognitive domains. Non-parametric statistics were used due to small sample size (Figure 3).

**Figure 3:**
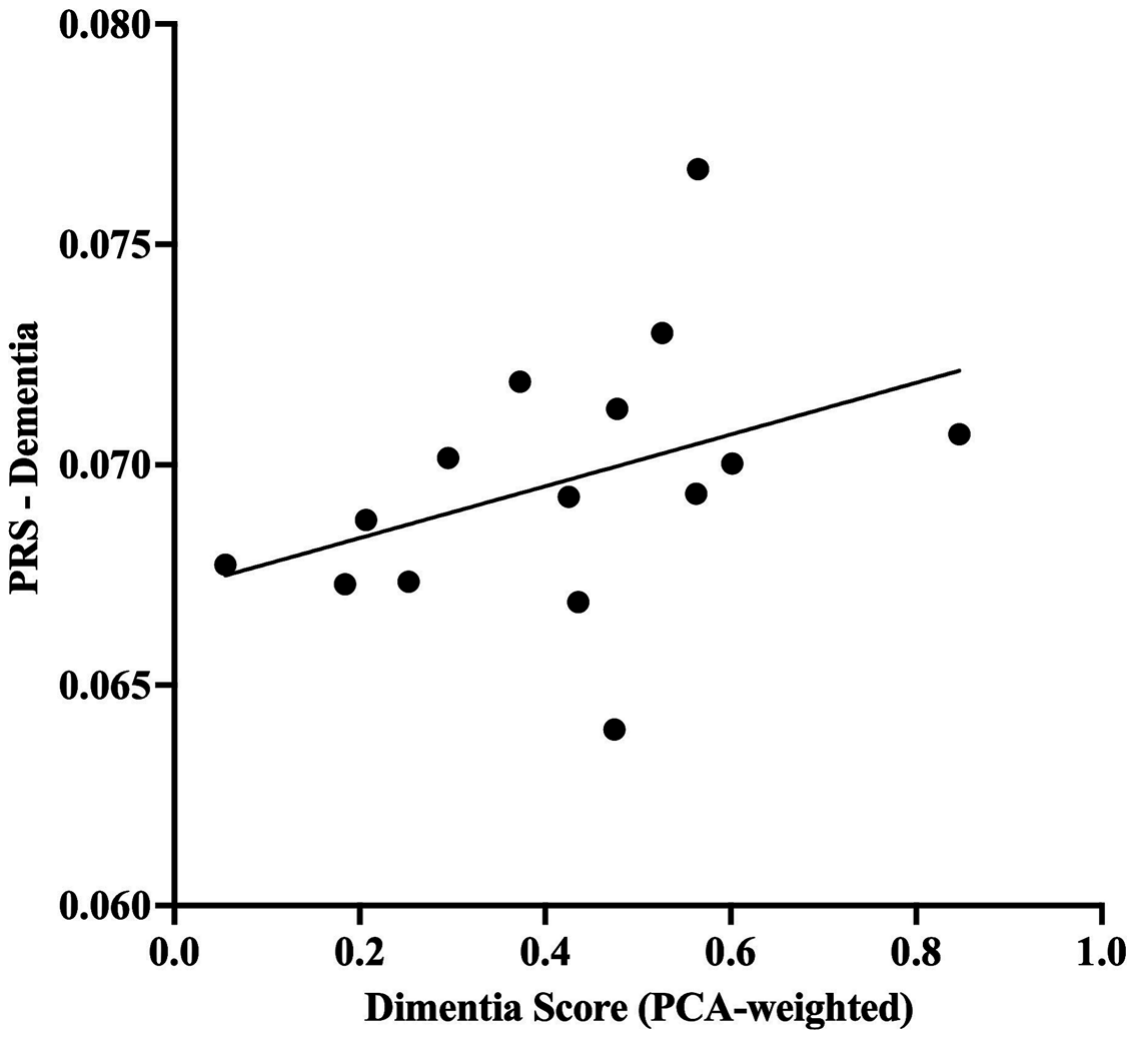
Exploratory association between PCA-weighted subjective cognitive burden and dementia-related polygenic risk score (PRS). Each point represents one of the 15 participants who completed the phenotype assessment. The fitted line is provided only to visualize the direction of the observed sample correlation and does not indicate a statistically established or validated association. The correlation estimate was Spearman’s r = 0.51 (95% CI −0.02 to 0.82; *p* = 0.054). Given the small sample size, wide confidence interval, unadjusted analysis, and possible selection bias, the figure should be interpreted as hypothesis-generating.

### 2.1 Polygenic risk score (PRS) estimation for dementia-related genetic susceptibility

The 60 individuals included in the present analysis were part of a previously recruited cohort described in detail elsewhere (15). These participants were adults (18–52 years) recruited from the state of Karnataka in India, belonging to a diverse ethnic background, between 2021 and 2023 under institutional ethics approvals (YEC-1/2021/034 and YEC-1/2023/236). Inclusion criteria required documented prior SARS-CoV-2 infection confirmed by reverse transcription polymerase chain reaction (RT-PCR) or rapid antigen testing (RAT) performed at certified diagnostic laboratories. Individuals with incomplete diagnostic documentation and those who did not take either of these two tests were excluded. All participants were recruited following recovery from the acute phase of infection. Clinical data, including age, sex, self-reported ancestry, and history of COVID-19 infection, were collected at enrollment. The present study represents a secondary genomic analysis of this previously characterized cohort (Table 1).

**Table 1:** Individual-level cognitive domain scores, dementia-weighted composite indices, and memory-specific PCA scores in COVID-19 patients included in the phenotype–genotype analysis.

Sr. no.	Age	Gender	Demography	Educational level	Dementia_clinical_weighted	Dementia_variance_weighted	Dementia_PCA_weighted
1	43	Male	Bengali	PhD	0.25833333	0.21039799	0.25251555
2	25	Female	Malayali	MSc	0.52083333	0.57563089	0.56257294
3	25	Female	Kannadiga	MSc	0.53333333	0.53316532	0.56482729
4	52	Male	Tulu	PhD	0.40833333	0.43901498	0.42520184
5	33	Male	Bengali	PhD	0.22916667	0.22693527	0.20669474
6	41	Male	Bengali	PhD	0.38333333	0.32281465	0.29510384
7	44	Female	Malayali	PhD	0.58333333	0.53888771	0.60153351
8	43	Female	Kannadiga	MSc	0.5125	0.44019446	0.4775634
9	30	Male	Bengali	MSc	0.44583333	0.39120379	0.37259977
10	30	Female	Malayali	MSc	0.36666667	0.37461919	0.43562848
11	43	Female	Punjabi	PhD	0.85416667	0.82108561	0.84632019
12	37	Male	Kannadiga	MSc	0.075	0.00858359	0.05505933
13	46	Female	Malayali	PhD	0.59166667	0.57011818	0.52627958
14	38	Female	Kannadiga	PhD	0.24166667	0.16712972	0.18425814
15	29	Female	Malayali	MSc	0.4875	0.47996119	0.4745767

To estimate polygenic risk scores (PRS), genome-wide genotype data from 60 COVID-19 patients (15) were merged with genome-wide data from 915 South Asian individuals obtained from the GenomeAsia100K project and our in-house laboratory database using PLINK v1.9. These 915 individuals were used as population reference samples and were not recruited as matched clinical controls for cognitive outcomes. They were either recruited to GenomeAsia100K prior to the COVID-19 pandemic or had no recorded history of SARS-CoV-2 infection at the time of sample collection; individuals with known COVID-19 diagnosis were excluded. Because the COVID-19 cohort and reference samples were not matched on genetic ancestry or clinical characteristics, the PRS comparison between groups is treated as descriptive and is not interpreted as a case–control association or evidence that COVID-19 status is genetically associated with dementia risk.

Genome-wide genotype data had undergone standard quality-control procedures in the original study (15), including SNP and sample call-rate filtering, Hardy–Weinberg equilibrium filtering, minor allele frequency filtering, removal of related individuals, and ancestry quality assessment. The present study used this post-QC dataset directly. Following merging with the reference dataset and strand harmonization, 527,110 overlapping single-nucleotide polymorphisms (SNPs) remained for PRS estimation. Ancestry quality assessment was therefore available from the original cohort QC; however, ancestry principal components were not included as covariates in the present phenotype–genotype analysis. This was a deliberate conservative choice given the very small cognitive subgroup (*n* = 15), in which multivariable adjustment would be unstable and prone to overfitting. The absence of PC adjustment is consequently treated as a limitation, particularly because the dementia base GWAS was predominantly European-ancestry whereas the target cohort comprised South Asian participants. APOE and MHC regions were retained in the primary PRS because the original objective was to estimate genome-wide genetic susceptibility. An APOE-exclusion sensitivity analysis was not performed in the present analysis and is therefore identified as an important direction for follow-up.

Polygenic risk scores (PRS) for dementia were generated using PRSice-2 (16) from the FinnGen all-cause dementia genome-wide association study (GWAS; finn-b-KRA_PSY_DEMENTIA), downloaded through the IEU OpenGWAS project. The source GWAS comprised 5,933 dementia cases and 212,859 controls and included 16,380,466 SNPs. Effect-size estimates (BETA) were used to construct additive PRS in the target dataset. Variants were restricted to those overlapping between the GWAS summary statistics and the target genotype dataset. LD clumping was performed using an r^2^ threshold of 0.1, a 250-kb window, and a clumping *p*-value threshold of 1.0. PRS were evaluated across GWAS *p*-value thresholds ranging from 5 × 10^−8^ to 0.5 in increments of 5 × 10^−5^. The best-fitting threshold identified by PRSice was pT = 0.00030005, corresponding to 190 SNPs, an explained variance (R^2^) of 0.064487, and a model *p*-value of 2.85 × 10^−11^ in the target analysis. The same PRS construction parameters were applied to the target dataset.

Because the threshold was selected through best-fit testing within the available dataset and no independent validation dataset or outcome-independent threshold-selection procedure was available, the resulting PRS was treated as an exploratory genetic score rather than a validated predictive model. The best-fit statistics were therefore not interpreted as evidence of clinical predictive performance, and no inference regarding individual dementia risk was made. Where PRS quartiles were used, the lowest quartile (bottom 25%) was treated as the reference category for descriptive comparison only; these quartiles were not interpreted as clinically validated risk categories.

Given that the base GWAS was derived predominantly from European-ancestry populations, interpretation of the PRS in the South Asian target population was considered in the context of potential limitations in cross-ancestry transferability (12). The dementia PRS was based on summary statistics from a predominantly European-ancestry GWAS of all-cause dementia susceptibility. It was not intended to represent current dementia status or serve as a clinically predictive score in the relatively young study cohort, but rather as an exploratory measure of inherited genetic liability potentially relevant to cognitive-related phenotypes.

### 2.2 Study participants and cognitive assessment

Of the 60 COVID-19–affected individuals, 15 consented to participate in the cognitive assessment component analysis and were included in phenotype–genotype correlation analyses. The initial cohort size (*n* = 60) was determined by availability of previously collected post-COVID genomic datasets generated under an earlier institutional study (15), rather than by a prospective power calculation. The present investigation was designed as an exploratory, hypothesis-generating secondary analysis integrating polygenic risk estimation with subjective cognitive phenotyping. Of the 60 eligible participants, all were approached for participation in the cognitive follow-up component. Fifteen individuals consented to complete the questionnaire-based assessment and were included in the phenotype–genotype correlation analysis, whereas the remaining participants were either unavailable during the follow-up period, declined participation, or could not be re-contacted. Because cognitive assessment participation was voluntary and the final sub-cohort was small, the possibility of selection bias cannot be excluded.

Cognitive symptoms were assessed 24–30 months following documented SARS-CoV-2 infection. All participants were evaluated after recovery from the acute phase, and none were assessed during active infection. Time since infection was not modeled as a covariate because of the small sample size and exploratory design; this heterogeneity is acknowledged as a potential source of variability. Importantly, the presence of a documented prior SARS-CoV-2 infection does not establish that subsequent subjective cognitive complaints were caused by COVID-19, because pre-infection cognitive status and several potentially relevant clinical and psychosocial factors were unavailable. The present study therefore evaluates cognitive complaints in individuals with a history of infection rather than attributing those complaints causally to COVID-19.

To evaluate whether the 15 individuals who completed the cognitive assessment differed systematically from the full COVID-19 cohort (*n* = 60), we compared key demographic and genetic characteristics between groups. Age distributions were compared using the Mann–Whitney U test, sex distribution using a chi-square test of independence, and standardized dementia-related PRS using the Mann–Whitney U test. These comparisons were descriptive and were not used to establish absence of selection bias. Non-significant results cannot exclude selection bias, particularly with only 15 participants in the cognitive subgroup. Two-tailed *p*-values were reported, with *p* < 0.05 used as a conventional descriptive threshold; effect estimates and uncertainty were emphasized over dichotomous significance.

Subjective cognitive function was evaluated using a structured, study-specific self-report questionnaire developed for the exploratory assessment of perceived everyday cognitive difficulties across memory, attention, executive functioning, learning, and language domains. The questionnaire was informed by commonly reported cognitive symptoms described in the post-COVID and subjective cognitive decline literature but is not a formally validated neuropsychological instrument. It was selected because the cognitive assessment was implemented as an exploratory follow-up component within an existing genomics cohort, rather than as a dedicated clinical neurocognitive evaluation. Responses were recorded in binary format, indicating the presence or absence of perceived cognitive difficulty, and were subsequently summarized quantitatively into domain-level and overall composite measures as described below. These measures were intended to capture subjective cognitive complaints and were not considered equivalent to objective neuropsychological test scores or diagnostic measures of cognitive impairment.

Internal consistency was examined using the Kuder–Richardson Formula 20 (KR-20), which is appropriate for binary items. Given the small sample size (*n* = 15) and sparse endorsement of several items, reliability estimates were considered unstable and are not used as evidence of formal instrument validity.

Objective neuropsychological testing was not performed. Standardized instruments such as the Montreal Cognitive Assessment (MoCA), Mini-Mental State Examination (MMSE), or formal memory-performance batteries were not administered. Accordingly, the cognitive outcomes should be interpreted strictly as subjective cognitive complaints. The study cannot determine whether reported complaints represent objective cognitive impairment, nor can it distinguish post-COVID effects from pre-existing symptoms or other factors such as fatigue, sleep disturbance, anxiety, mood, or stress.

### 2.3 Cognitive domain construction

Individual questionnaire items were grouped into theoretically informed cognitive domains based on established neuropsychological constructs and prior dementia literature. Domain assignment was performed *a priori* by the investigators before statistical analysis and was not data-driven. The domains and representative examples are outlined below:

#### 2.3.1 Memory domain

Items reflecting episodic and working memory difficulties, such as forgetting names of familiar people or places, difficulty recalling recent conversations, forgetting passwords, and difficulty remembering lists of items just read or heard.

#### 2.3.2 Executive function domain

Items capturing planning, task execution, and cognitive flexibility, including difficulty applying newly learned methods, forgetting administrative steps or procedures, and missing scheduled meetings.

#### 2.3.3 Attention domain

Items related to sustained and divided attention, such as losing track of tasks after short interruptions and needing to repeatedly review procedures already performed multiple times.

#### 2.3.4 Learning domain

Items assessing acquisition of new information or skills, including difficulty remembering new software commands and needing more repetitions when learning something new.

#### 2.3.5 Language domain

Items reflecting word retrieval and semantic access difficulties, such as difficulty recalling technical terms or abbreviations.

For each participant, domain scores were calculated as the mean of item responses within each domain, with higher scores indicating greater perceived cognitive difficulty. The questionnaire was not treated as a validated neuropsychological scale and was intended only to summarize subjective cognitive experiences.

We note her that no missing values were present for the variables included in each individual analysis; however, the number of participants varied across analyses according to the availability of the corresponding phenotypic or assessment data. In particular, the exploratory cognitive analysis included only participants who completed the relevant cognitive assessment.

### 2.4 Derivation of dementia-weighted cognitive scores

To derive a global summary of subjective cognitive burden, principal component analysis (PCA) was performed on the five domain scores (Memory, Executive, Attention, Learning, and Language) in R v4.4.1. PCA was used strictly as a dimensionality-reduction technique to summarize covariance among the five self-reported domains and was not interpreted as evidence of a latent disease process or clinical impairment. The first principal component (PC1) was retained as a descriptive summary of the largest shared variance component. Domain scores were weighted using their PC1 loadings to compute a PCA-weighted cognitive score. Because *n* = 15 is small for PCA, the loadings and the 52.5% variance explained should be considered sample-specific and potentially unstable rather than generalizable estimates. Also, given the small number of individuals available for the cognitive subgroup (*n* = 15), PCA was used only for exploratory visualization of genetic variation. The resulting patterns were not interpreted as definitive evidence of population structure or ancestry differentiation. No item-level missing data were present, so no items were excluded or imputed for missingness. A memory-specific PCA score was also derived from the memory-related questionnaire items as an exploratory secondary summary.

### 2.5 Polygenic risk score correlation

Dementia-related PRS were available for each individual. The association between the PCA-weighted cognitive score and the corresponding dementia PRS was evaluated using Spearman’s rank correlation in GraphPad Prism v10. Spearman’s correlation was selected because of the small sample size and the non-normal distribution of the cognitive scores. Two-tailed *p*-values and 95% confidence intervals were reported. No multivariable adjustment for age, sex, education, time since infection, COVID-19 severity, psychosocial factors, or ancestry principal components was performed because the cognitive subgroup contained only 15 participants.

Notably, genetic principal component analysis was performed to characterize ancestry structure. However, ancestry principal components were not included as covariates in the PRS comparison or phenotype–PRS correlation analyses because these analyses were intended as exploratory descriptive comparisons rather than ancestry-adjusted association models. The analysis therefore should be treated as unadjusted and hypothesis-generating rather than as a definitive association model (18).

## 3 Results

All results presented below are exploratory and intended to generate hypotheses rather than establish causal or predictive relationships.

### 3.1 Comparison of cognitive subset with the full COVID-19 cohort

To determine whether the cognitive sub-cohort (*n* = 15) differed from the full COVID-19 cohort (*n* = 60), we compared demographic and genetic characteristics between groups. Age distribution did not differ significantly between the two groups (Mann–Whitney U = 310.0, *p* = 0.1045), although the sub-cohort showed a modest trend toward higher median age (38 vs. 32 years). Sex distribution was comparable (χ^2^ = 0.0352, *p* = 0.8512), and standardized dementia-related PRS did not differ significantly between groups (Mann–Whitney U = 364.0, *p* = 0.3002). These non-significant comparisons do not demonstrate equivalence and cannot rule out selection bias; with *n* = 15, the study had limited power to detect modest differences.

Taken together, the observed demographic and PRS distributions did not show large differences between the cognitive subgroup and the full COVID-19 cohort on the measured variables. However, these comparisons cannot establish the absence of selection bias because the subgroup was small and participation was voluntary. Unmeasured differences, including clinical and psychosocial characteristics, may remain.

### 3.2 Comparison of PRS for dementia between the COVID-19 patients and other south Asians

A descriptive comparison of dementia-related polygenic risk scores (PRS) was performed between individuals with a history of COVID-19 infection and South Asian population reference samples included in the merged genotype dataset (Supplementary Table 1). COVID-19–affected individuals showed a modest rightward shift in standardized PRS distribution compared with reference samples, with substantial overlap between groups (mean standardized PRS: 0.24 vs. − 0.01; Mann–Whitney U = 29,504.5, *p* = 0.065; Cohen’s d = 0.25). We note here that while the COVID-19 history group showed a modestly higher mean standardized dementia PRS than the population-reference group, the difference should be interpreted as a descriptive, exploratory observation rather than evidence of increased inherited dementia susceptibility. This small distributional difference should not be interpreted as evidence of increased dementia susceptibility, because the groups were not matched clinical controls and residual differences in genetic ancestry and other characteristics may contribute to the observed PRS distribution. The result is therefore presented as a descriptive pattern requiring validation in ancestry-matched samples.

### 3.3 Cognitive domain structure and PCA loadings

Principal component analysis (PCA) was applied to five self-reported cognitive domains to summarize shared variance across domains. PC1 accounted for 52.5% of the observed variance and was used as a descriptive composite of subjective cognitive burden. Memory had the largest loading (0.555), followed by Learning (0.511), Executive function (0.493), Language (0.365), and Attention (0.235). The loading pattern suggests that memory-related complaints contributed most strongly to the shared variance in this sample. Because the PCA was based on only 15 participants and a study-specific questionnaire, the loadings should be interpreted as exploratory, sample-specific estimates rather than stable population parameters.

### 3.4 Memory-specific cognitive scores

Memory-specific PCA scores were derived for all 15 participants from responses to memory-related questionnaire items (Supplementary Tables 2, 3). Item-level missingness was 0% across all questionnaire items; therefore, no items were excluded or imputed because of missing data. Owing to limited variance and sparse endorsement patterns, internal consistency estimates were unstable and are not emphasized. The resulting scores summarize patterns of self-reported memory difficulty and are used only as exploratory descriptive measures.

To summarize subjective cognitive burden across domains, three complementary composite indices were derived. First, a clinical-weighted score was calculated as the unweighted mean of domain scores. Second, a variance-weighted score was calculated by weighting each domain according to its relative contribution to overall variance. Third, a PCA-weighted score was derived by multiplying each standardized domain score by its PC1 loading and summing the resulting values. The PCA-weighted composite was used as the primary dimensionality-reduction measure for exploratory correlation analysis. All composite measures are study-specific exploratory indices and do not represent validated clinical diagnostic scales.

### 3.5 Association between cognitive burden and dementia polygenic risk

Exploratory correlation analysis was performed to examine the relationship between dementia-related PRS and PCA-weighted subjective cognitive burden among the 15 participants who completed cognitive assessment. Spearman’s rank correlation was *r* = 0.51 (95% CI − 0.02 to 0.82; *p* = 0.054). The effect estimate is compatible with a positive association, but the confidence interval is wide and includes values close to no association, reflecting substantial uncertainty in this small sample. The result is therefore reported as an exploratory pattern rather than a statistically conclusive finding and does not imply predictive or causal association.

## 4 Discussion

In this brief research report, we investigated whether polygenic susceptibility captured by a dementia-related PRS was associated with subjective cognitive burden among individuals with a history of COVID-19. We observed a modest distributional difference in PRS between the COVID-19 cohort and South Asian population reference samples and an exploratory positive correlation between PRS and perceived cognitive burden in the *n* = 15 cognitive subgroup. The reference-sample comparison is descriptive rather than a matched case–control analysis, and the phenotype–genotype association is severely underpowered. Accordingly, the findings are best viewed as preliminary evidence motivating larger, appropriately controlled studies rather than as evidence of increased dementia risk or a clinically meaningful predictive relationship.

Persistent cognitive complaints following coronavirus disease 2019 (COVID-19) have increasingly been recognized as a core component of post-acute sequelae of SARS-CoV-2 infection (PASC). Multiple studies and systematic reviews have documented impairments in memory, attention, executive function, and overall cognitive clarity in individuals months after recovery, including those who experienced mild or non-hospitalized disease (2). However, the severity, duration, and specific cognitive domains affected vary substantially across individuals, indicating that host-specific biological factors may contribute to post-COVID cognitive vulnerability.

Cognitive traits such as memory and learning are highly complex and polygenic, shaped by the cumulative effects of numerous common genetic variants of small effect. Large-scale genome-wide association studies (GWAS) have demonstrated significant heritability for both general cognitive ability and domain-specific cognitive traits, supporting a distributed genetic architecture underlying cognitive performance (3, 4, 7). In particular, GWAS meta-analyses have identified multiple loci associated with verbal short-term memory and learning, highlighting the substantial genetic contribution to memory-related processes (6). These findings provide a strong biological rationale for investigating whether background genetic susceptibility may influence cognitive outcomes following systemic stressors such as viral infection.

Polygenic risk scores (PRS) provide a quantitative framework for summarizing cumulative genetic susceptibility derived from GWAS summary statistics. In the context of neurodegenerative disease, PRS for Alzheimer’s disease and related dementias have been shown to associate with cognitive performance, cognitive decline, and neurobiological markers even among individuals without clinical dementia (9–11). Importantly, PRS are not deterministic and typically explain only a modest proportion of phenotypic variance. Their utility lies primarily in population-level inference and hypothesis generation rather than individual-level prediction (13).

The observed rightward shift in dementia-related PRS among individuals with a history of COVID-19 should be interpreted cautiously. The substantial overlap between groups, *p* = 0.065, and small Cohen’s d of 0.25 indicate a modest distributional difference rather than evidence of increased dementia susceptibility. Because the population reference samples were not matched clinical controls and the groups may differ in genetic ancestry and other characteristics, the comparison cannot establish that COVID-19 status is associated with higher inherited dementia risk. We note here that the comparison with population-reference participants was not a matched case–control analysis and should not be interpreted as demonstrating that COVID-19 is associated with increased inherited susceptibility to dementia. Differences between groups in recruitment context, demographic composition, ancestry structure, or other unmeasured factors may influence PRS distributions. The observed difference is therefore considered exploratory and hypothesis-generating.

Integration of the self-reported cognitive domains using PCA showed that memory-related difficulties had the largest loading on PC1. However, given the small sample size (*n* = 15), the PCA pattern should be regarded as descriptive and sample-specific, potentially sensitive to sampling variation. PCA was used for exploratory dimensionality reduction and visualization, not for formal ancestry inference or identification of latent neurodegenerative or clinical constructs.

The phenotype–genotype analysis yielded a Spearman correlation of *r* = 0.51 (95% CI − 0.02 to 0.82; *p* = 0.054), indicating a positive effect estimate but substantial uncertainty. This estimate should not be interpreted as evidence of a statistically established or predictive association. The small sample size, possible selection bias, absence of multivariable adjustment, lack of objective cognitive testing, and use of a predominantly European-derived dementia GWAS in a South Asian cohort all limit inference. Future studies should test whether the observed direction and magnitude persist after adjustment for demographic, clinical, ancestry, and genetic factors in larger cohorts.

A plausible biological framework linking polygenic dementia susceptibility to post-COVID cognitive symptoms involves immune-mediated mechanisms. Genetic risk for dementia, particularly late-onset Alzheimer’s disease, has been shown to implicate pathways related to neuroinflammation, microglial activation, lipid metabolism, and innate immune signaling. SARS-CoV-2 infection induces systemic inflammatory responses and cytokine dysregulation, which may transiently or persistently affect central nervous system function. It is therefore conceivable that individuals with higher polygenic susceptibility to neurodegenerative processes may exhibit heightened vulnerability to inflammation-triggered cognitive disturbances following infection. While the present study does not directly assess immune markers, this hypothesis provides a biologically coherent framework for future mechanistic investigations integrating genomics, inflammatory profiling, and longitudinal cognitive assessment.

Several limitations should be considered when interpreting these findings. First, the primary phenotype–genotype analysis included only 15 participants and is therefore severely underpowered; the observed correlation should be regarded as exploratory and hypothesis-generating, with the wide confidence interval indicating considerable uncertainty around the estimate.

Second, participation in the cognitive follow-up was voluntary, and non-significant comparisons with the full cohort cannot rule out selection bias. While baseline characteristics were compared between participants with and without cognitive assessments, non-significant statistical tests do not establish subgroup representativeness or rule out systematic differences, particularly given the small sample size. Participation in cognitive assessment may have been influenced by health status, availability, willingness to participate, or other unmeasured factors. Consequently, findings from the *n* = 15 subgroup may not generalize to the broader COVID-19 history cohort.

Third, cognitive complaints were assessed using a study-specific, non-validated self-report questionnaire rather than standardized neuropsychological testing. Thus, the outcomes reflect subjective cognitive burden rather than objectively measured cognitive impairment or diagnostic status. Responses may also be influenced by fatigue, sleep quality, anxiety, mood, stress, recall, and other psychosocial factors. Although several cognitive domains were assessed and internal consistency was evaluated where appropriate, future studies should incorporate validated cognitive screening tools and objective neuropsychological assessments.

Fourth, the cross-sectional design and absence of pre-infection cognitive measurements limit conclusions regarding the temporal relationship between COVID-19 and the reported cognitive complaints and prevent assessment of whether similar complaints were present before infection.

Fifth, the dementia PRS was derived from a predominantly European-ancestry all-cause dementia GWAS, which may reduce transferability to South Asian participants. No independent replication sample with matched post-COVID cognitive and genotype data was available, so the reported association remains hypothesis-generating. Also, the source GWAS assessed genetic susceptibility to all-cause dementia, a phenotype primarily studied in older populations, and therefore its relevance to subjective cognitive complaints in this relatively young cohort is uncertain. The PRS should consequently be interpreted as an exploratory genetic liability measure rather than a dementia-risk prediction tool or indicator of clinical dementia.

Another important limitation is that several potentially relevant demographic, clinical, lifestyle, and genetic confounders were either unavailable, incompletely captured, or not included in multivariable adjustment. Age, sex, education, time since infection, COVID-19 severity, fatigue, sleep, psychological factors, and genetic principal components may influence subjective cognitive burden or PRS interpretation. Because the cognitive subgroup contained only 15 participants, including multiple covariates would risk unstable and overfit models. We therefore used an unadjusted non-parametric correlation as a conservative exploratory analysis. This choice should not be interpreted as evidence that confounding is absent. Future studies with substantially larger, ancestry-matched samples should prespecify multivariable models incorporating relevant demographic, clinical, and genetic covariates.

Population stratification also remains an important limitation. While genetic PCA was used to characterize ancestry structure, ancestry principal components were not included as covariates in the PRS analyses. Consequently, residual ancestry-related structure may have contributed to the observed PRS distributions or phenotype–PRS relationships, and the findings should not be interpreted as ancestry-adjusted genetic associations. Larger studies with greater representation and more detailed modelling of ancestry structure will be required to evaluate whether these observations persist after appropriate adjustment.

Finally, an APOE-specific sensitivity analysis was not performed because the cognitive subgroup was limited to 15 participants, making additional covariate adjustment vulnerable to unstable estimates and further loss of statistical power; moreover, the relevance and cross-ancestry transferability of APOE-related effects for this phenotype in South Asian populations require further evaluation.

The interval between SARS-CoV-2 infection and cognitive assessment ranged from 24 to 30 months. Although all participants were assessed after the acute infection period, this variation may contribute to heterogeneity in subjective symptoms. Because time since infection was not modeled in the present small sample, longitudinal designs with standardized assessment intervals are needed.

The cognitive sub-cohort consisted predominantly of individuals with postgraduate education. Educational attainment may influence cognitive reserve, symptom awareness, and reporting of subjective difficulties, limiting generalizability to populations with different educational backgrounds.

Despite these limitations, the study demonstrates the feasibility of integrating genome-wide genotype data, GWAS-derived PRS, and structured subjective cognitive phenotyping in a post-COVID cohort. The strengths include genome-wide genotype data, standardized PRS construction, an understudied South Asian population, and an explicitly hypothesis-generating analytical framework. The study also identifies methodological priorities for future work: ancestry-matched PRS development or calibration, objective neuropsychological assessment, adequate sample size, longitudinal follow-up, prespecified covariate adjustment, independent replication, and sensitivity analyses examining the contribution of APOE.

## 5 Conclusion

Overall, this exploratory study identified a modest distributional difference in dementia-related PRS between individuals with a history of COVID-19 and South Asian population reference samples and a positive but uncertain association between dementia-related PRS and subjective cognitive burden in a small cognitive subgroup. These findings should not be interpreted as evidence that COVID-19 increases inherited dementia risk, that the cognitive complaints were caused by COVID-19, or that the PRS can predict individual cognitive outcomes. The primary phenotype–genotype result is severely underpowered, potentially affected by selection and residual population stratification, and based on a non-validated subjective outcome without multivariable adjustment. Larger, ancestry-matched, longitudinal studies incorporating standardized neuropsychological testing, clinical covariates, independent replication, and APOE sensitivity analyses are required to determine whether the observed exploratory pattern is reproducible.

## Data Availability

The original contributions presented in the study are included in the article/Supplementary material, further inquiries can be directed to the corresponding author.
