# Supplementary material for: Potential association between dementia polygenic risk scores and subjective cognitive burden in a post-COVID cohort: a hypothesis-generating study
Source: Front Public Health. 2026 Oct 1;14:1867492. doi: 10.3389/fpubh.2026.1867492 (PMC13669090; doi:10.3389/fpubh.2026.1867492)
Supplement: Supplementary file 1 [file Table_1.DOCX]

**Supplementary Table 1**: Dementia PRS scores for both COVID patients and population control

FID	IID	In_Regression	PRS

Kerala	207762980012_R01C01	Yes	0.075773947

Kerala	207762980038_R01C01	Yes	0.073434211

Kerala	207762980012_R01C02	Yes	0.073042895

Kerala	207762980038_R01C02	Yes	0.071356579

Kerala	207762980012_R02C01	Yes	0.074723158

Kerala	207762980038_R02C01	Yes	0.075541579

Kerala	207762980012_R02C02	Yes	0.076786842

Kerala	207762980038_R02C02	Yes	0.067100526

Kerala	207762980012_R03C01	Yes	0.066837895

Kerala	207762980038_R03C01	Yes	0.066821316

Kerala	207762980008_R03C02	Yes	0.070251014

Kerala	207762980012_R03C02	Yes	0.074920263

Kerala	207762980038_R03C02	Yes	0.072943421

Kerala	207762980008_R04C01	Yes	0.069469474

Kerala	207762980012_R04C01	Yes	0.067373947

Kerala	207762980038_R04C01	Yes	0.075567105

Kerala	207762980008_R04C02	Yes	0.068911987

Kerala	207762980012_R04C02	Yes	0.07520079

Kerala	207762980038_R04C02	Yes	0.076443421

Kerala	207762980008_R05C01	Yes	0.073970263

Kerala	207762980012_R05C01	Yes	0.072589737

Kerala	207762980038_R05C01	Yes	0.068352632

Kerala	207762980004_R05C02	Yes	0.072052105

Kerala	207762980008_R05C02	Yes	0.074893249

Kerala	207762980012_R05C02	Yes	0.075241579

Kerala	207762980038_R05C02	Yes	0.071599211

Kerala	207762980004_R06C01	Yes	0.076919474

Kerala	207762980008_R06C01	Yes	0.068422196

Kerala	207762980012_R06C01	Yes	0.070751316

Kerala	207762980038_R06C01	Yes	0.066145086

Kerala	207762980004_R06C02	Yes	0.076933421

Kerala	207762980008_R06C02	Yes	0.075587459

Kerala	207762980012_R06C02	Yes	0.07269

Kerala	207762980038_R06C02	Yes	0.075593158

Kerala	207762980004_R07C01	Yes	0.068379737

Kerala	207762980008_R07C01	Yes	0.074869211

Kerala	207762980012_R07C01	Yes	0.071282368

Kerala	207762980038_R07C01	Yes	0.076804474

Kerala	207762980004_R07C02	Yes	0.070568684

Kerala	207762980008_R07C02	Yes	0.06859088

Kerala	207762980012_R07C02	Yes	0.072665263

Kerala	207762980038_R07C02	Yes	0.074634737

Kerala	207762980004_R08C01	Yes	0.064997535

Kerala	207762980008_R08C01	Yes	0.074411579

Kerala	207762980012_R08C01	Yes	0.069356053

Kerala	207762980038_R08C01	Yes	0.07444579

Kerala	207762980004_R08C02	Yes	0.076972632

Kerala	207762980008_R08C02	Yes	0.072863775

Kerala	207762980012_R08C02	Yes	0.076492895

Kerala	207762980038_R08C02	Yes	0.074341316

Kerala	207762980004_R09C01	Yes	0.072849737

Kerala	207762980008_R09C01	Yes	0.076476579

Kerala	207762980012_R09C01	Yes	0.072460855

Kerala	207762980038_R09C01	Yes	0.075131842

Kerala	207762980004_R09C02	Yes	0.070084737

Kerala	207762980008_R09C02	Yes	0.068976143

Kerala	207762980012_R09C02	Yes	0.073604737

Kerala	207762980038_R09C02	Yes	0.076901842

Kerala	207762980004_R10C01	Yes	0.070845

Kerala	207762980008_R10C01	Yes	0.074732895

Kerala	207762980012_R10C01	Yes	0.069167105

Kerala	207762980038_R10C01	Yes	0.07530079

Kerala	207762980004_R10C02	Yes	0.076163684

Kerala	207762980008_R10C02	Yes	0.074223683

Kerala	207762980012_R10C02	Yes	0.068483947

Kerala	207762980038_R10C02	Yes	0.07206579

Kerala	207762980004_R11C01	Yes	0.068322324

Kerala	207762980008_R11C01	Yes	0.065616894

Kerala	207762980012_R11C01	Yes	0.075712105

Kerala	207762980038_R11C01	Yes	0.076923158

Kerala	207762980004_R11C02	Yes	0.069583947

Kerala	207762980008_R11C02	Yes	0.073236523

Kerala	207762980012_R11C02	Yes	0.069573511

Kerala	207762980038_R11C02	Yes	0.07599

Kerala	207762980004_R12C01	Yes	0.076947895

Kerala	207762980008_R12C01	Yes	0.06820754

Kerala	207762980012_R12C01	Yes	0.071017236

Kerala	207762980038_R12C01	Yes	0.076475621

Kerala	207762980004_R12C02	Yes	0.073382895

Kerala	207762980008_R12C02	Yes	0.067728684

Kerala	207762980012_R12C02	Yes	0.077076579

Kerala	207762980038_R12C02	Yes	0.070326579

ABM	GA000856	Yes	0.068269737

ABM	GA000857	Yes	0.067288684

ABM	GA000858	Yes	0.063245263

ABM	GA000859	Yes	0.065584474

ABM	GA000860	Yes	0.052321053

ABM	GA000861	Yes	0.062486842

ABM	GA000862	Yes	0.064631579

ABM	GA000863	Yes	0.066595263

ABM	GA000864	Yes	0.065047368

ABM	GA000865	Yes	0.073367632

ABM	GA000866	Yes	0.061147632

AGH	GA000808	Yes	0.058077105

AGH	GA000809	Yes	0.062713947

AGH	GA000810	Yes	0.069731053

AGH	GA000811	Yes	0.066225

AGH	GA000812	Yes	0.064098421

AGH	GA000813	Yes	0.066805526

AGH	GA000814	Yes	0.070532368

AGH	GA000815	Yes	0.070579211

AGH	GA000816	Yes	0.067685526

AGH	GA000817	Yes	0.070226579

AGH	GA000818	Yes	0.065267758

AGH	GA000819	Yes	0.071015074

AGH	GA000820	Yes	0.064955

AGH	GA000821	Yes	0.070521442

AGH	GA000822	Yes	0.072104863

AGH	GA000823	Yes	0.070968396

AGH	GA000867	Yes	0.068662548

Northeast_Karnataka	Ankylosis_9111470	Yes	0.073821316

Northeast_Karnataka	Ankylosis_9111483	Yes	0.072418134

Northeast_Karnataka	Ankylosis_9111487	Yes	0.072393158

Northeast_Karnataka	Ankylosis_R01C02	Yes	0.063030996

Northeast_Karnataka	Ankylosis_R02C02	Yes	0.063030996

Northeast_Karnataka	Ankylosis_R03C02	Yes	0.058203864

Northeast_Karnataka	Ankylosis_R04C02	Yes	0.063655121

Northeast_Karnataka	Ankylosis_R05C02	Yes	0.071152499

Northeast_Karnataka	Ankylosis_R06C02	Yes	0.072107473

Northeast_Karnataka	Ankylosis_R07C02	Yes	0.06864105

Northeast_Karnataka	Ankylosis_R12C01	Yes	0.069935683

Coastal_Karnataka_Kerala	ARDS_8948829	Yes	0.068544449

Coastal_Karnataka_Kerala	ARDS_8961977	Yes	0.068715979

Coastal_Karnataka_Kerala	ARDS_8961978	Yes	0.062501579

Coastal_Karnataka_Kerala	ARDS_8961981	Yes	0.063679713

Coastal_Karnataka_Kerala	ARDS_8961984	Yes	0.071175263

Coastal_Karnataka_Kerala	ARDS_8961985	Yes	0.070418684

Coastal_Karnataka_Kerala	ARDS_8961987	Yes	0.073127632

Coastal_Karnataka_Kerala	ARDS_9045801	Yes	0.060404474

Coastal_Karnataka_Kerala	ARDS_9045802	Yes	0.071742829

Coastal_Karnataka_Kerala	ARDS_9045803	Yes	0.069005526

Coastal_Karnataka_Kerala	ARDS_9157949	Yes	0.073267607

Coastal_Karnataka_Kerala	ARDS_9157950	Yes	0.071782895

Coastal_Karnataka_Kerala	ARDS_9157952	Yes	0.071589211

Coastal_Karnataka_Kerala	ARDS_9157956	Yes	0.06927722

Coastal_Karnataka_Kerala	ARDS_9157959	Yes	0.063679211

Coastal_Karnataka_Kerala	ARDS_9157965	Yes	0.068996579

Coastal_Karnataka_Kerala	ARDS_9157967	Yes	0.066602282

Assam	7639166	Yes	0.06630002

BAG	GA000870	Yes	0.062588495

BAG	GA000871	Yes	0.05991381

BAG	GA000872	Yes	0.061014337

COVID	7639164	Yes	0.068426842

COVID	7639167	Yes	0.071883428

COVID	7639168	Yes	0.066062873

COVID	7639170	Yes	0.068748158

COVID	7639173	Yes	0.06840182

COVID	7639174	Yes	0.070154474

COVID	7639176	Yes	0.070466579

COVID	7819779	Yes	0.067347933

BHM	GA000893	Yes	0.066207231

BHM	GA000896	Yes	0.062785

BHM	GA000897	Yes	0.069629211

BHM	GA000899	Yes	0.06520079

BHM	GA000900	Yes	0.06362579

BIR	GA000873	Yes	0.072973684

BIR	GA000874	Yes	0.068802105

BIR	GA000875	Yes	0.067177105

BIR	GA000876	Yes	0.066049474

BIR	GA000877	Yes	0.064743421

BIR	GA000878	Yes	0.066151579

BIR	GA000879	Yes	0.061977368

BIR	GA000880	Yes	0.064083421

BIR	GA000881	Yes	0.07071

BIR	GA000882	Yes	0.064694211

BIR	GA000883	Yes	0.06354579

BIR	GA000884	Yes	0.070622632

BIR	GA000885	Yes	0.062007632

BIR	GA000886	Yes	0.055403947

BIR	GA000887	Yes	0.064100526

BIR	GA000888	Yes	0.066583947

BIR	GA000889	Yes	0.059898684

BIR	GA000890	Yes	0.068829474

BIR	GA000891	Yes	0.068616316

BIR	GA000892	Yes	0.069824737

BLR	GA001274	Yes	0.070198947

BLR	GA001275	Yes	0.066579211

BLR	GA001276	Yes	0.063138947

BLR	GA001277	Yes	0.067125

BLR	GA001278	Yes	0.072433684

BLR	GA001279	Yes	0.071567632

BLR	GA001280	Yes	0.06535579

BLR	GA001281	Yes	0.072093947

BLR	GA001282	Yes	0.063803684

BLR	GA001284	Yes	0.061513421

BLR	GA001285	Yes	0.074034737

BLR	GA001286	Yes	0.075009737

BLR	GA001287	Yes	0.068723158

BLR	GA001288	Yes	0.071570747

BLR	GA001289	Yes	0.069736316

BLR	GA001292	Yes	0.071926842

BLR	GA001295	Yes	0.074665526

BLR	GA001296	Yes	0.067337632

BLR	GA001298	Yes	0.065999211

BLR	GA001299	Yes	0.071314211

BLR	GA001305	Yes	0.068013158

BLR	GA001307	Yes	0.070694474

BLR	GA001308	Yes	0.06134579

BLR	GA001309	Yes	0.068685526

BLR	GA001311	Yes	0.058514211

BLR	GA001312	Yes	0.071768421

BLR	GA001313	Yes	0.065236579

BLR	GA001314	Yes	0.069135526

BLR	GA001316	Yes	0.066379211

BLR	GA001319	Yes	0.065287632

BLR	GA001320	Yes	0.06147579

BLR	GA001321	Yes	0.065880263

BLR	GA001322	Yes	0.064731316

BLR	GA001323	Yes	0.066188421

BRA	GA001826	Yes	0.073440263

BRA	GA001827	Yes	0.072551842

BRA	GA001828	Yes	0.064681053

BRA	GA001829	Yes	0.072924211

BRA	GA001830	Yes	0.06353579

BRA	GA001831	Yes	0.069966316

BRA	GA001832	Yes	0.066288158

BRA	GA001834	Yes	0.064163684

BRA	GA001835	Yes	0.068258421

BRU	GA001837	Yes	0.063972105

BRU	GA001838	Yes	0.069256842

BRU	GA001839	Yes	0.067631842

BRU	GA001865	Yes	0.066065263

BRU	GA001866	Yes	0.071286579

BRU	GA001867	Yes	0.071398158

BRU	GA001869	Yes	0.065786579

BRU	GA001871	Yes	0.066792105

COVID	7819789	Yes	0.069891842

CHK	GA000994	Yes	0.057925

CHK	GA000995	Yes	0.060156842

CHK	GA000996	Yes	0.064819474

CHK	GA000997	Yes	0.060958947

CHK	GA000998	Yes	0.058938421

CHK	GA000999	Yes	0.065013421

CHK	GA001000	Yes	0.067291316

CHK	GA001001	Yes	0.072942105

CHK	GA001002	Yes	0.071073684

CHK	GA001003	Yes	0.052198947

CHK	GA001004	Yes	0.064936681

CHM	GA000963	Yes	0.063733613

CHM	GA000964	Yes	0.067495263

CHM	GA000965	Yes	0.065610526

CHM	GA000966	Yes	0.072599211

CHM	GA000967	Yes	0.070125765

CHM	GA000968	Yes	0.061704863

CHN	GA001074	Yes	0.067942368

CHN	GA001075	Yes	0.069249211

CHN	GA001076	Yes	0.055626842

CHN	GA001077	Yes	0.064333158

CHN	GA001078	Yes	0.065175723

CHN	GA001079	Yes	0.062068947

CHN	GA001080	Yes	0.066466842

CHN	GA001081	Yes	0.066613947

Coastal_Karnataka_Kerala	7988729	Yes	0.07050579

Coastal_Karnataka_Kerala	7988730	Yes	0.074530565

Coastal_Karnataka_Kerala	7988731	Yes	0.074786537

Coastal_Karnataka_Kerala	7988732	Yes	0.070354474

Coastal_Karnataka_Kerala	7988733	Yes	0.06273079

Coastal_Karnataka_Kerala	7988734	Yes	0.072793158

Coastal_Karnataka_Kerala	7988737	Yes	0.062601486

Coastal_Karnataka_Kerala	7988738	Yes	0.07581895

Coastal_Karnataka_Kerala	7988739	Yes	0.077242632

Coastal_Karnataka_Kerala	7988740	Yes	0.065942514

Coastal_Karnataka_Kerala	7988741	Yes	0.06690579

Coastal_Karnataka_Kerala	7988742	Yes	0.065824934

Coastal_Karnataka_Kerala	7988743	Yes	0.069538421

Coastal_Karnataka_Kerala	7988744	Yes	0.064893421

Coastal_Karnataka_Kerala	7988745	Yes	0.066682829

Coastal_Karnataka_Kerala	7988746	Yes	0.06399

Coastal_Karnataka_Kerala	7988747	Yes	0.071431274

Coastal_Karnataka_Kerala	7988748	Yes	0.069832895

Coastal_Karnataka_Kerala	7988749	Yes	0.068200526

Coastal_Karnataka_Kerala	7988750	Yes	0.066821312

Coastal_Karnataka_Kerala	7988752	Yes	0.063164737

Coastal_Karnataka_Kerala	7988753	Yes	0.066330445

Coastal_Karnataka_Kerala	7988754	Yes	0.061314737

Coastal_Karnataka_Kerala	7988755	Yes	0.066924452

Coastal_Karnataka_Kerala	7988756	Yes	0.070803793

Coastal_Karnataka_Kerala	7988757	Yes	0.066067276

Coastal_Karnataka_Kerala	7988758	Yes	0.072319474

Coastal_Karnataka_Kerala	7988759	Yes	0.06782079

Coastal_Karnataka_Kerala	7988760	Yes	0.070533684

Coastal_Karnataka_Kerala	7988761	Yes	0.064990433

Coastal_Karnataka_Kerala	8307215	Yes	0.065004211

Coastal_Karnataka_Kerala	8307216	Yes	0.064070526

Coastal_Karnataka_Kerala	8307217	Yes	0.066969118

Coastal_Karnataka_Kerala	8307218	Yes	0.06698079

Coastal_Karnataka_Kerala	8307220	Yes	0.067175263

Coastal_Karnataka_Kerala	8307221	Yes	0.064301053

Coastal_Karnataka_Kerala	8307223	Yes	0.066344129

Coastal_Karnataka_Kerala	8307226	Yes	0.065245263

Coastal_Karnataka_Kerala	8307227	Yes	0.069814186

Coastal_Karnataka_Kerala	8405021	Yes	0.06695579

Coastal_Karnataka_Kerala	8405022	Yes	0.064589121

Coastal_Karnataka_Kerala	8405024	Yes	0.069532105

Coastal_Karnataka_Kerala	8405025	Yes	0.065912895

Coastal_Karnataka_Kerala	8405026	Yes	0.06555812

Coastal_Karnataka_Kerala	8415942	Yes	0.055428127

Coastal_Karnataka_Kerala	8415943	Yes	0.066545708

Coastal_Karnataka_Kerala	8415944	Yes	0.070184737

Coastal_Karnataka_Kerala	R01C01	Yes	0.072403781

Coastal_Karnataka_Kerala	R02C01	Yes	0.067458421

DHR	GA000824	Yes	0.06160079

DHR	GA000825	Yes	0.067086316

DHR	GA000826	Yes	0.071908421

DHR	GA000827	Yes	0.068177105

DHR	GA000828	Yes	0.067615526

DHR	GA000829	Yes	0.06814579

DHR	GA000830	Yes	0.058561579

DHR	GA000831	Yes	0.068353158

DHR	GA000832	Yes	0.064702895

DHR	GA000833	Yes	0.069825

DOR	GA000834	Yes	0.060891316

DOR	GA000835	Yes	0.066028684

DOR	GA000836	Yes	0.066960263

DOR	GA000837	Yes	0.065453158

DOR	GA000838	Yes	0.068386053

DOR	GA000839	Yes	0.07404

DOR	GA000840	Yes	0.06411

DOR	GA000841	Yes	0.061011579

DOR	GA000842	Yes	0.064238684

DOR	GA000843	Yes	0.071987368

DOR	GA000844	Yes	0.06356

DOR	GA000845	Yes	0.067967632

GAU	GA001052	Yes	0.072155263

GAU	GA001053	Yes	0.071606579

GAU	GA001054	Yes	0.069538158

GAU	GA001055	Yes	0.065773947

GAU	GA001056	Yes	0.069375526

GAU	GA001057	Yes	0.066057632

GAU	GA001058	Yes	0.066479211

GAU	GA001059	Yes	0.06732079

GAU	GA001060	Yes	0.063087105

GUJ	GA001792	Yes	0.06867079

GUJ	GA001793	Yes	0.070890263

GUJ	GA001794	Yes	0.069031842

GUJ	GA001795	Yes	0.067560526

GUJ	GA001796	Yes	0.069429474

GUJ	GA001797	Yes	0.072261842

GUJ	GA001798	Yes	0.060055

GUJ	GA001799	Yes	0.064331316

GUJ	GA001800	Yes	0.067927105

GUJ	GA001801	Yes	0.060914474

GUJ	GA001802	Yes	0.065641316

GUJ	GA001803	Yes	0.06894079

GUJ	GA001804	Yes	0.062934211

GUJ	GA001805	Yes	0.064059474

GUJ	GA001806	Yes	0.067706842

GUJ	GA001807	Yes	0.074418684

GUJ	GA001808	Yes	0.070838947

GUJ	GA001809	Yes	0.070539474

GUJ	GA001810	Yes	0.063401053

GUJ	GA001811	Yes	0.070618947

COVID	7639175	Yes	0.074336818

HAZ	GA001873	Yes	0.070110526

HAZ	GA001874	Yes	0.070015263

HAZ	GA001875	Yes	0.064094211

HAZ	GA001876	Yes	0.063620526

HAZ	GA001877	Yes	0.064106579

HAZ	GA001878	Yes	0.071264474

HAZ	GA001880	Yes	0.064309211

HAZ	GA001881	Yes	0.068324737

HAZ	GA001882	Yes	0.067742368

HAZ	GA001883	Yes	0.061755

HAZ	GA001884	Yes	0.062604211

HAZ	GA001885	Yes	0.068409211

HAZ	GA001886	Yes	0.064296053

HAZ	GA001887	Yes	0.070417895

HAZ	GA001888	Yes	0.068351053

COVID	7639169	Yes	0.068354907

COVID	7639184	Yes	0.07240182

COVID	7819780	Yes	0.070696431

HKR	GA000901	Yes	0.069459211

HKR	GA000902	Yes	0.067601842

HKR	GA000903	Yes	0.05683079

HKR	GA000904	Yes	0.070373684

HKR	GA000905	Yes	0.061347632

HKR	GA000906	Yes	0.067196842

HKR	GA000907	Yes	0.064451316

HKR	GA000908	Yes	0.065628684

HKR	GA000909	Yes	0.062371842

HKR	GA000910	Yes	0.062443947

HLB	GA001061	Yes	0.059833158

HLB	GA001188	Yes	0.069203158

HLB	GA001189	Yes	0.062547632

HLB	GA001190	Yes	0.067669211

HLB	GA001191	Yes	0.068048158

HLB	GA001192	Yes	0.066607105

IRU	GA000632	Yes	0.067442895

IRU	GA000633	Yes	0.071943684

ITU	GA001399	Yes	0.069392895

ITU	GA001400	Yes	0.069611842

ITU	GA001401	Yes	0.054318421

ITU	GA001408	Yes	0.073723684

ITU	GA001409	Yes	0.070231842

ITU	GA001410	Yes	0.065181579

ITU	GA001411	Yes	0.06777079

ITU	GA001412	Yes	0.066692895

ITU	GA001413	Yes	0.064662632

ITU	GA001414	Yes	0.071540263

ITU	GA001415	Yes	0.061438684

ITU	GA001416	Yes	0.073529737

ITU	GA001417	Yes	0.068717368

ITU	GA001418	Yes	0.064915526

ITU	GA001419	Yes	0.063577105

ITU	GA001420	Yes	0.067936053

ITU	GA001421	Yes	0.067028947

ITU	GA001422	Yes	0.069460263

ITU	GA001423	Yes	0.066597895

ITU	GA001424	Yes	0.069012632

ITU	GA001425	Yes	0.066041579

ITU	GA001426	Yes	0.069172895

ITU	GA001427	Yes	0.070150263

ITU	GA001428	Yes	0.062200263

ITU	GA001429	Yes	0.070077105

IYA	GA001082	Yes	0.069905263

IYA	GA001083	Yes	0.070888421

IYA	GA001085	Yes	0.063028547

IYA	GA001086	Yes	0.070313158

IYA	GA001087	Yes	0.059285652

IYA	GA001088	Yes	0.069462105

IYE	GA001089	Yes	0.065319737

IYE	GA001090	Yes	0.068012895

IYE	GA001091	Yes	0.075252632

IYE	GA001092	Yes	0.071478158

IYE	GA001093	Yes	0.073331842

IYE	GA001094	Yes	0.074503421

IYE	GA001095	Yes	0.067897996

IYE	GA001096	Yes	0.071732632

IYE	GA001097	Yes	0.071008684

IYE	GA001098	Yes	0.074001842

IYE	GA001099	Yes	0.058005

IYE	GA001100	Yes	0.071673158

IYE	GA001101	Yes	0.073006842

JAM	GA001005	Yes	0.066225

JAM	GA001006	Yes	0.066166316

JAM	GA001007	Yes	0.06757079

JAM	GA001008	Yes	0.057594737

JAM	GA001009	Yes	0.067733158

JAM	GA001010	Yes	0.067588158

JAM	GA001011	Yes	0.061216053

JAM	GA001012	Yes	0.064201053

JAM	GA001013	Yes	0.057622632

JAR	GA000781	Yes	0.064921053

JAR	GA000782	Yes	0.064792368

JAR	GA000783	Yes	0.055944737

JAR	GA000784	Yes	0.063134211

JAR	GA000785	Yes	0.069176316

JAR	GA000786	Yes	0.065511579

JAR	GA000787	Yes	0.058530526

JAR	GA000788	Yes	0.071156316

JAR	GA000789	Yes	0.069074211

JAR	GA000790	Yes	0.074128684

JAR	GA000791	Yes	0.061331316

JAR	GA000792	Yes	0.05923

KAM	GA000912	Yes	0.067221053

KAM	GA000913	Yes	0.067208684

KAM	GA000914	Yes	0.066763947

KAM	GA000915	Yes	0.07328079

KAM	GA000916	Yes	0.068686053

KAM	GA000917	Yes	0.066870526

KAM	GA000918	Yes	0.071118684

KAM	GA000919	Yes	0.065087632

KAM	GA000920	Yes	0.063553158

KAM	GA000921	Yes	0.069966053

KAM	GA000922	Yes	0.064732632

COVID	7639157	Yes	0.066296274

COVID	7639158	Yes	0.070358947

COVID	7639172	Yes	0.067732105

COVID	7819788	Yes	0.064681555

COVID	8076550	Yes	0.068715623

COVID	8076551	Yes	0.071298397

KHA	GA000969	Yes	0.068557895

KHA	GA000970	Yes	0.072084474

KHA	GA000971	Yes	0.077899211

KHA	GA000972	Yes	0.068401579

KHA	GA000973	Yes	0.067924737

KHA	GA000974	Yes	0.068628684

KHA	GA000975	Yes	0.071503421

KHA	GA000976	Yes	0.062863684

KHA	GA000977	Yes	0.066061053

KHA	GA000978	Yes	0.070664211

KHA	GA000979	Yes	0.070375526

KNR	GA001113	Yes	0.06209

KNR	GA001114	Yes	0.066236842

KNR	GA001115	Yes	0.073092632

KNR	GA001116	Yes	0.071178684

KNR	GA001117	Yes	0.06650079

KNR	GA001118	Yes	0.065556579

KNR	GA001119	Yes	0.074643158

KNR	GA001120	Yes	0.060332758

KNR	GA001121	Yes	0.06204079

KNR	GA001122	Yes	0.065654474

KNR	GA001123	Yes	0.061379737

KNR	GA001124	Yes	0.070791316

KNR	GA001125	Yes	0.061630263

KNR	GA001126	Yes	0.066244211

KNR	GA001127	Yes	0.058728158

KNR	GA001128	Yes	0.067037632

KNR	GA001129	Yes	0.061842632

COVID	7639154	Yes	0.068912895

COVID	7639159	Yes	0.070572895

COVID	7639177	Yes	0.068700787

COVID	7819790	Yes	0.074972607

COVID	8076549	Yes	0.061516579

Koraga	7485829	Yes	0.068082933

Koraga	7485830	Yes	0.076693158

Koraga	7485831	Yes	0.06652517

Koraga	7485832	Yes	0.070726053

Koraga	7485833	Yes	0.070978684

Koraga	7485835	Yes	0.067435971

Koraga	7485836	Yes	0.069022347

Koraga	7485838	Yes	0.069526053

Koraga	7485839	Yes	0.066652632

Koraga	7485840	Yes	0.07343579

Koraga	7485841	Yes	0.066065316

Koraga	7485842	Yes	0.068930971

Koraga	7485843	Yes	0.065851761

Koraga	7485844	Yes	0.064352105

Koraga	7485846	Yes	0.073150526

Koraga	7485848	Yes	0.071129211

Koraga	7485849	Yes	0.065224737

Koraga	B2	Yes	0.068164474

Koraga	B3	Yes	0.071590785

Koraga	B4	Yes	0.066512105

Koraga	B5	Yes	0.064494211

Koraga	B6	Yes	0.06381579

Koraga	B7	Yes	0.064543136

Koraga	B9	Yes	0.072566235

Koraga	B10	Yes	0.067417948

Koraga	B11	Yes	0.061314439

Koraga	B12	Yes	0.067600708

Koraga	B14	Yes	0.061927368

Koraga	B17	Yes	0.069382526

KTA	GA001179	Yes	0.066186053

KTA	GA001180	Yes	0.068197105

KTA	GA001181	Yes	0.063751053

KTA	GA001182	Yes	0.06626579

KTA	GA001183	Yes	0.067581579

KTA	GA001184	Yes	0.070256818

KTA	GA001186	Yes	0.072688133

KTA	GA001187	Yes	0.070606053

KYD	GA001102	Yes	0.070677157

KYD	GA001103	Yes	0.067376442

KYD	GA001104	Yes	0.065164989

KYD	GA001105	Yes	0.069486842

KYD	GA001106	Yes	0.060757494

KYD	GA001107	Yes	0.072214211

KYD	GA001108	Yes	0.067753947

KYD	GA001109	Yes	0.069109863

KYD	GA001110	Yes	0.074155263

KYD	GA001111	Yes	0.068911842

KYD	GA001112	Yes	0.062238947

Lakshadweep	201334790059_R01C01	Yes	0.073159001

Lakshadweep	201334790059_R01C02	Yes	0.067197632

Lakshadweep	201334790059_R02C01	Yes	0.066532632

Lakshadweep	201334790059_R02C02	Yes	0.068661579

Lakshadweep	201334790059_R03C01	Yes	0.069030866

Lakshadweep	201334790059_R03C02	Yes	0.074302041

Lakshadweep	201334790059_R04C01	Yes	0.065031053

Lakshadweep	201334790059_R04C02	Yes	0.070253684

Lakshadweep	201334790059_R05C01	Yes	0.065325526

Lakshadweep	201334790059_R05C02	Yes	0.067197632

Lakshadweep	201334790059_R06C01	Yes	0.07091

Lakshadweep	201334790059_R06C02	Yes	0.066094211

Lakshadweep	201334790059_R07C01	Yes	0.072186053

Lakshadweep	201334790059_R07C02	Yes	0.06974

Lakshadweep	201334790059_R08C01	Yes	0.075889474

Lakshadweep	201334790059_R08C02	Yes	0.06974

Lakshadweep	201334790059_R09C01	Yes	0.069660415

Lakshadweep	201334790059_R09C02	Yes	0.068361316

Lakshadweep	201334790059_R10C01	Yes	0.064902632

Lakshadweep	201334790059_R10C02	Yes	0.068120477

Lakshadweep	201334790059_R11C01	Yes	0.066446807

Lakshadweep	201334790059_R11C02	Yes	0.064479337

Lakshadweep	201334790059_R12C01	Yes	0.074848158

Lakshadweep	201334790059_R12C02	Yes	0.068572914

Lakshadweep	201334790061_R01C01	Yes	0.069619307

Lakshadweep	201334790061_R01C02	Yes	0.070143684

Lakshadweep	201334790061_R02C01	Yes	0.061623094

Lakshadweep	201334790061_R02C02	Yes	0.061873635

Lakshadweep	201334790061_R03C01	Yes	0.068501442

Lakshadweep	201334790061_R03C02	Yes	0.061728158

Lakshadweep	201334790061_R04C01	Yes	0.071351053

Lakshadweep	201334790061_R04C02	Yes	0.064688158

Lakshadweep	201334790061_R05C01	Yes	0.075745

Lakshadweep	201334790061_R05C02	Yes	0.071408395

Lakshadweep	201334790061_R06C01	Yes	0.070614211

Lakshadweep	201334790061_R06C02	Yes	0.072769205

Lakshadweep	201334790061_R07C01	Yes	0.063605

Lakshadweep	201334790061_R07C02	Yes	0.064047368

Lakshadweep	201334790061_R08C01	Yes	0.072775263

Lakshadweep	201334790061_R08C02	Yes	0.074642559

Lakshadweep	201334790061_R09C01	Yes	0.068232368

Lakshadweep	201334790061_R09C02	Yes	0.066611053

Lakshadweep	201334790061_R10C01	Yes	0.067842959

Lakshadweep	201334790061_R10C02	Yes	0.067287988

Lakshadweep	201334790061_R11C01	Yes	0.070038947

Lakshadweep	201334790061_R11C02	Yes	0.07103903

Lakshadweep	201334790061_R12C01	Yes	0.06742167

LAM	GA001130	Yes	0.064055

LAM	GA001131	Yes	0.061956316

LAM	GA001132	Yes	0.058465

LAM	GA001133	Yes	0.06688079

LAM	GA001134	Yes	0.066756842

LAM	GA001135	Yes	0.067861579

LAM	GA001136	Yes	0.068274211

LAM	GA001137	Yes	0.069333158

LAM	GA001138	Yes	0.063277632

LAM	GA001139	Yes	0.066518421

LAM	GA001140	Yes	0.066958947

LAM	GA001141	Yes	0.064903421

LAM	GA001142	Yes	0.061004737

LAM	GA001143	Yes	0.070939474

LAM	GA001144	Yes	0.063432105

LAM	GA001145	Yes	0.060288947

LAM	GA001146	Yes	0.069737368

LOD	GA000923	Yes	0.069062895

LOD	GA000924	Yes	0.073158947

LOD	GA000925	Yes	0.066443947

LOD	GA000926	Yes	0.063520263

LOD	GA000927	Yes	0.068299474

LOD	GA000928	Yes	0.072242632

LOD	GA000929	Yes	0.071480263

LOD	GA000930	Yes	0.072368158

LOD	GA000931	Yes	0.068536579

LOD	GA000932	Yes	0.064325526

LOD	GA000933	Yes	0.061378158

MAA	GA001229	Yes	0.066411316

MAA	GA001230	Yes	0.066203158

MAA	GA001231	Yes	0.074899737

MAA	GA001232	Yes	0.07106579

MAA	GA001234	Yes	0.066478421

MAA	GA001240	Yes	0.070724737

MAA	GA001241	Yes	0.063692105

MAA	GA001242	Yes	0.070448421

MAA	GA001243	Yes	0.067442105

MAA	GA001244	Yes	0.074094211

MAA	GA001245	Yes	0.065781316

MAA	GA001246	Yes	0.069609474

MAA	GA001247	Yes	0.06728

MAA	GA001248	Yes	0.064802632

MAA	GA001251	Yes	0.065439211

MAA	GA001252	Yes	0.065107368

MAA	GA001253	Yes	0.065048947

MAA	GA001254	Yes	0.069185263

MAA	GA001256	Yes	0.07423079

MAA	GA001257	Yes	0.072297632

MAA	GA001258	Yes	0.073541579

MAA	GA001259	Yes	0.068919474

MAA	GA001260	Yes	0.067980526

MAA	GA001262	Yes	0.061884211

MAA	GA001263	Yes	0.06598579

MAA	GA001264	Yes	0.07051079

MAA	GA001265	Yes	0.05806579

MAA	GA001266	Yes	0.062413421

MAA	GA001267	Yes	0.065333684

MAA	GA001268	Yes	0.075126579

MAA	GA001269	Yes	0.066818947

MAA	GA001271	Yes	0.068457632

MAA	GA001272	Yes	0.062902368

MAA	GA001273	Yes	0.074503684

COVID	7639155	Yes	0.071129757

COVID	7639156	Yes	0.062166842

COVID	7639162	Yes	0.067289656

COVID	7639165	Yes	0.072992105

COVID	7639171	Yes	0.069443642

COVID	7639181	Yes	0.069341842

COVID	7639183	Yes	0.062905526

COVID	7819775	Yes	0.066883662

COVID	7819776	Yes	0.073614186

COVID	7819778	Yes	0.063992105

COVID	7819782	Yes	0.068897632

COVID	7819783	Yes	0.074722895

COVID	7819791	Yes	0.070030809

COVID	7819792	Yes	0.068834715

COVID	7874277	Yes	0.068538421

COVID	7874279	Yes	0.069871579

COVID	8076546	Yes	0.066676842

COVID	8076548	Yes	0.066033938

COVID	8076553	Yes	0.069957105

COVID	7639179	Yes	0.056635526

MHR	GA001193	Yes	0.075055263

MHR	GA001195	Yes	0.063826579

MHR	GA001196	Yes	0.070835526

MHR	GA001197	Yes	0.063616316

MHR	GA001198	Yes	0.066701053

MHR	GA001199	Yes	0.069762368

MHR	GA001200	Yes	0.066392895

MHR	GA001201	Yes	0.065543421

MHR	GA001202	Yes	0.065371179

MHR	GA001203	Yes	0.064775

MHR	GA001204	Yes	0.075226053

MHR	GA001205	Yes	0.07000579

MHR	GA001206	Yes	0.071521316

MHR	GA001207	Yes	0.076638021

MHR	GA001208	Yes	0.069065

MHR	GA001209	Yes	0.064641842

MHR	GA001210	Yes	0.065722632

MHR	GA001211	Yes	0.071268421

MHR	GA001212	Yes	0.061142758

MNP	GA001014	Yes	0.062352948

MNP	GA001016	Yes	0.065557136

MNP	GA001017	Yes	0.06268

MNP	GA001018	Yes	0.066001552

MNP	GA001019	Yes	0.064286316

MOG	GA001020	Yes	0.069419474

MOG	GA001021	Yes	0.064473684

MOG	GA001022	Yes	0.062493158

MOG	GA001023	Yes	0.063323421

MOG	GA001024	Yes	0.066153947

MOG	GA001025	Yes	0.060932895

MOG	GA001026	Yes	0.067473947

MOG	GA001027	Yes	0.065144737

MOG	GA001028	Yes	0.07113579

MOG	GA001029	Yes	0.061421316

MOG	GA001030	Yes	0.061091579

MOG	GA001031	Yes	0.065857368

MOG	GA001032	Yes	0.066182632

MOG	GA001033	Yes	0.062869211

MOG	GA001034	Yes	0.070553158

MOG	GA001035	Yes	0.061516053

MOG	GA001037	Yes	0.062952368

MOG	GA001038	Yes	0.062228421

MOG	GA001039	Yes	0.069746316

MOG	GA001040	Yes	0.068542368

MUN	GA000934	Yes	0.067674474

MUN	GA000935	Yes	0.058455

MUR	GA000846	Yes	0.070691848

MUR	GA000847	Yes	0.064456842

MUR	GA000848	Yes	0.06034

MUR	GA000849	Yes	0.069107105

MUR	GA000850	Yes	0.062354737

MUR	GA000851	Yes	0.067393947

MUR	GA000852	Yes	0.067029474

MUR	GA000853	Yes	0.07035079

MUR	GA000854	Yes	0.066345526

MUR	GA000855	Yes	0.062992895

NAB	GA001214	Yes	0.072329474

NAB	GA001215	Yes	0.071538421

NAB	GA001217	Yes	0.071320526

NAB	GA001218	Yes	0.064408684

NIC	GA001890	Yes	0.066650263

NIC	GA001891	Yes	0.067682895

NIC	GA001892	Yes	0.071035

NIC	GA001893	Yes	0.067526579

NIC	GA001894	Yes	0.064498421

NIC	GA001895	Yes	0.070546842

NRI	1_Sample_1	Yes	0.074461705

NRI	2_Sample_2	Yes	0.06878866

NRI	3_Sample_3	Yes	0.072376179

NRI	4_Sample_4	Yes	0.06366948

NRI	5_Sample_5	Yes	0.062485515

NRI	6_Sample_6	Yes	0.060119737

NRI	7_Sample_7	Yes	0.073228947

NRI	8_Sample_8	Yes	0.063661818

NRI	9_Sample_9	Yes	0.067467924

NRI	10_Sample_10	Yes	0.067042039

NRI	11_Sample_11	Yes	0.075798427

NRI	12_Sample_12	Yes	0.062861059

NRI	13_Sample_13	Yes	0.056588473

NRI	14_Sample_14	Yes	0.073262501

NRI	15_Sample_16	Yes	0.059989099

NRI	16_Sample_18	Yes	0.056092071

NRI	17_Sample_19	Yes	0.075142896

NRI	18_Sample_20	Yes	0.074036659

NRI	19_Sample_21	Yes	0.063848631

NRI	20_Sample_22	Yes	0.071017262

NRI	21_Sample_23	Yes	0.069714017

NRI	22_Sample_24	Yes	0.069527925

ONG	GA000793	Yes	0.058881842

ONG	GA000794	Yes	0.061855526

ONG	GA000795	Yes	0.062235

ONG	GA000796	Yes	0.05408579

ONG	GA000797	Yes	0.054316053

ONG	GA000798	Yes	0.053060526

ONG	GA000799	Yes	0.056676842

ONG	GA000800	Yes	0.060167895

ONG	GA000801	Yes	0.057093684

ONG	GA000802	Yes	0.058995

ONG	GA000803	Yes	0.055264474

ONG	GA000804	Yes	0.059712368

ONG	GA000805	Yes	0.066456316

ONG	GA000806	Yes	0.068471842

ONG	GA000807	Yes	0.068837105

ORN	GA000936	Yes	0.066409474

ORN	GA000937	Yes	0.065297632

ORN	GA000938	Yes	0.061169211

ORN	GA000939	Yes	0.066880526

ORN	GA000940	Yes	0.069747105

ORN	GA000941	Yes	0.066054211

ORN	GA000942	Yes	0.069951842

ORN	GA000943	Yes	0.06739

ORN	GA000944	Yes	0.067465263

ORN	GA000945	Yes	0.06121

ORN	GA000946	Yes	0.068998421

ORN	GA000947	Yes	0.062519211

ORN	GA000948	Yes	0.070088158

ORN	GA000949	Yes	0.074777368

ORN	GA000950	Yes	0.066829211

PAR	GA001872	Yes	0.069522105

PAT	GA001841	Yes	0.067604211

PAT	GA001842	Yes	0.068613947

PAT	GA001843	Yes	0.065076053

PAT	GA001844	Yes	0.070766316

PAT	GA001845	Yes	0.071317368

PAT	GA001846	Yes	0.068921316

PAT	GA001847	Yes	0.061258684

PAT	GA001848	Yes	0.070283158

PAT	GA001849	Yes	0.069199211

PAT	GA001850	Yes	0.072562632

PAT	GA001851	Yes	0.070784211

PAT	GA001852	Yes	0.070841579

PAT	GA001853	Yes	0.066836316

PAT	GA001854	Yes	0.065928684

PAT	GA001855	Yes	0.071474737

Coastal_Karnataka_Kerala	Pneumonia_8948830	Yes	0.055943092

Coastal_Karnataka_Kerala	Pneumonia_8948831	Yes	0.07084787

Coastal_Karnataka_Kerala	Pneumonia_8948835	Yes	0.073728947

Coastal_Karnataka_Kerala	Pneumonia_8948838	Yes	0.065524211

Coastal_Karnataka_Kerala	Pneumonia_8948839	Yes	0.073346028

Coastal_Karnataka_Kerala	Pneumonia_8948854	Yes	0.075634449

Coastal_Karnataka_Kerala	Pneumonia_8961979	Yes	0.06934787

Coastal_Karnataka_Kerala	Pneumonia_8961980	Yes	0.069616555

Coastal_Karnataka_Kerala	Pneumonia_8961982	Yes	0.065556053

Coastal_Karnataka_Kerala	Pneumonia_8961983	Yes	0.067026842

Coastal_Karnataka_Kerala	Pneumonia_8961986	Yes	0.073286028

Coastal_Karnataka_Kerala	Pneumonia_8961988	Yes	0.061852746

Coastal_Karnataka_Kerala	Pneumonia_8961989	Yes	0.070451579

Coastal_Karnataka_Kerala	Pneumonia_8961990	Yes	0.067409186

Coastal_Karnataka_Kerala	Pneumonia_9045796	Yes	0.067470433

Coastal_Karnataka_Kerala	Pneumonia_9045797	Yes	0.063446053

Coastal_Karnataka_Kerala	Pneumonia_9045798	Yes	0.064874538

Coastal_Karnataka_Kerala	Pneumonia_9045799	Yes	0.069918052

Coastal_Karnataka_Kerala	Pneumonia_9045800	Yes	0.072738684

PNY	GA001147	Yes	0.071397368

PNY	GA001148	Yes	0.058806842

PNY	GA001149	Yes	0.071914211

PNY	GA001150	Yes	0.068008158

PNY	GA001151	Yes	0.065608158

PNY	GA001152	Yes	0.06886079

PNY	GA001154	Yes	0.063186842

PNY	GA001155	Yes	0.060154474

PNY	GA001156	Yes	0.071141316

PNY	GA001157	Yes	0.067185526

PNY	GA001158	Yes	0.064542105

RAJ	GA001812	Yes	0.070414474

RAJ	GA001813	Yes	0.067446579

RAJ	GA001814	Yes	0.069183947

RAJ	GA001815	Yes	0.078353684

RAJ	GA001816	Yes	0.067355263

RAJ	GA001817	Yes	0.060978158

RAJ	GA001818	Yes	0.072328158

RAJ	GA001819	Yes	0.067544737

RAJ	GA001820	Yes	0.069241579

RAJ	GA001821	Yes	0.067358158

RAJ	GA001822	Yes	0.070456053

RAJ	GA001823	Yes	0.06928579

RAJ	GA001824	Yes	0.067424737

RAJ	GA001825	Yes	0.060952105

RTH	GA001062	Yes	0.066092368

RTH	GA001063	Yes	0.052525263

RTH	GA001064	Yes	0.06950079

RTH	GA001065	Yes	0.065503158

RTH	GA001066	Yes	0.065039474

RTH	GA001067	Yes	0.065636842

RTH	GA001068	Yes	0.06558079

RTH	GA001069	Yes	0.065414474

RTH	GA001070	Yes	0.067310263

RTH	GA001071	Yes	0.062421053

RTH	GA001072	Yes	0.071155526

RTH	GA001073	Yes	0.071049474

SND	GA001836	Yes	0.060027105

SND	GA001856	Yes	0.062387895

SND	GA001857	Yes	0.073707632

SND	GA001858	Yes	0.070292105

SND	GA001859	Yes	0.069749474

SND	GA001860	Yes	0.068745

SND	GA001861	Yes	0.069466316

SND	GA001862	Yes	0.074365526

SND	GA001863	Yes	0.063429737

SND	GA001864	Yes	0.066297632

SOB	GA001219	Yes	0.070131588

SOB	GA001220	Yes	0.07106324

SOB	GA001221	Yes	0.069547895

SOB	GA001222	Yes	0.069039094

SOB	GA001223	Yes	0.073929474

SOB	GA001224	Yes	0.068367632

SOB	GA001225	Yes	0.070275138

SOB	GA001227	Yes	0.07171579

SOB	GA001228	Yes	0.066164211

SRB	GA000980	Yes	0.067902368

SRB	GA000981	Yes	0.071427632

SRB	GA000982	Yes	0.068102895

SRB	GA000983	Yes	0.061554737

SRB	GA000984	Yes	0.071353947

SRB	GA000985	Yes	0.06539579

SRB	GA000986	Yes	0.072246316

SRB	GA000987	Yes	0.068285

SRB	GA000988	Yes	0.066217895

SRB	GA000989	Yes	0.073598684

SRB	GA000990	Yes	0.06659579

SRB	GA000991	Yes	0.076285263

SRB	GA000992	Yes	0.071658158

SRB	GA000993	Yes	0.067598947

STU	GA001397	Yes	0.064767368

STU	GA001398	Yes	0.071330526

STU	GA001402	Yes	0.072859474

STU	GA001403	Yes	0.068453947

STU	GA001404	Yes	0.069144211

STU	GA001405	Yes	0.066894211

STU	GA001406	Yes	0.072349737

STU	GA001407	Yes	0.070703158

SZH	GA000634	Yes	0.068135526

SZH	GA000635	Yes	0.069123947

SZH	GA000636	Yes	0.066974474

SZH	GA000637	Yes	0.063842105

SZH	GA000638	Yes	0.070243684

SZH	GA000639	Yes	0.067757105

SZH	GA000640	Yes	0.071567368

SZH	GA000641	Yes	0.068271053

SZH	GA000642	Yes	0.070479737

COVID	7639178	Yes	0.068768925

TNT	GA000951	Yes	0.062374211

TOD	GA001159	Yes	0.062303421

TOD	GA001160	Yes	0.067282895

TOD	GA001161	Yes	0.062043421

TOD	GA001162	Yes	0.061208158

TOD	GA001163	Yes	0.060943421

TOD	GA001164	Yes	0.055897632

TOD	GA001165	Yes	0.067276316

TOD	GA001166	Yes	0.070728947

TOD	GA001167	Yes	0.060418421

TOD	GA001168	Yes	0.068282368

TOD	GA001169	Yes	0.066582105

TOD	GA001170	Yes	0.058911579

TOD	GA001171	Yes	0.061979211

TOD	GA001172	Yes	0.066832105

TOD	GA001173	Yes	0.075531053

TOD	GA001174	Yes	0.067825

TOD	GA001175	Yes	0.065257895

TOD	GA001176	Yes	0.060585263

TOD	GA001177	Yes	0.059426579

TOD	GA001178	Yes	0.066073684

TTO	GA001041	Yes	0.061241316

TTO	GA001042	Yes	0.057547895

TTO	GA001043	Yes	0.058861842

TTO	GA001044	Yes	0.064049211

TTO	GA001045	Yes	0.063338947

TTO	GA001046	Yes	0.066322895

TTO	GA001047	Yes	0.068456579

TTO	GA001048	Yes	0.060796842

TTO	GA001049	Yes	0.058923684

TTO	GA001050	Yes	0.06554

TTO	GA001051	Yes	0.062217105

COVID	7639160	Yes	0.071263947

COVID	7639161	Yes	0.060608906

COVID	7639163	Yes	0.070940748

COVID	7819777	Yes	0.076708603

COVID	7819784	Yes	0.067586314

COVID	7874276	Yes	0.069270526

COVID	7874278	Yes	0.06356

COVID	7819787	Yes	0.064486798

COVID	8076545	Yes	0.079209474

COVID	8076547	Yes	0.068865545

COVID	8076552	Yes	0.064436842

WBB	GA000952	Yes	0.068553633

WBB	GA000953	Yes	0.059280526

WBB	GA000954	Yes	0.073594474

WBB	GA000955	Yes	0.065777134

WBB	GA000956	Yes	0.064778421

WBB	GA000957	Yes	0.062423947

WBB	GA000958	Yes	0.066157368

WBB	GA000959	Yes	0.071652632

WBB	GA000960	Yes	0.072680526

WBB	GA000961	Yes	0.06713299

**Supplementary Table 2**: Dementia Weighted Scores of 15 recruited participants
